# Supplementary figures and images for: Genome-Wide Identification and Characterization of the Soybean Snf2 Gene Family and Expression Response to Rhizobia
Source: Int J Mol Sci. 2023 Apr 14;24(8):7250. doi: 10.3390/ijms24087250 (PMC10138738; doi:10.3390/ijms24087250)

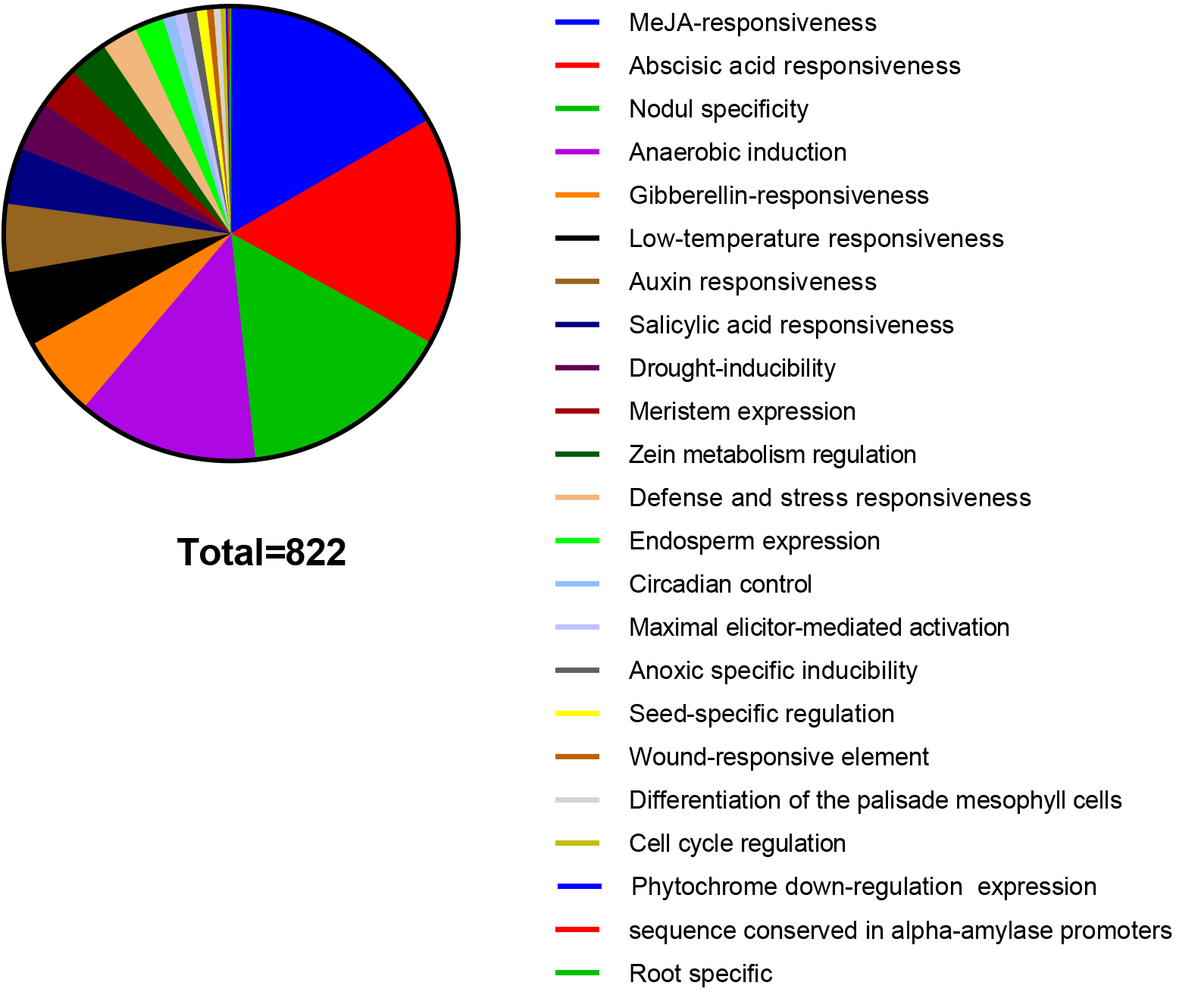

Supplement: Supplementary file 1 [file ijms-24-07250-s001.zip › Figure S1 The proportion of various cis-elements.jpg]
